# A coupling approach of a predictor and a descriptor for breast cancer prognosis

**DOI:** 10.1186/1755-8794-7-S1-S4

**Published:** 2014-05-08

**Authors:** Hyunjung Shin, Yonghyun Nam

**Affiliations:** 1 Department of Industrial Engineering, Ajou University, Wonchun-dong, Yeongtong-gu, Suwon 443-749, South Korea

## Abstract

**Background:**

In cancer prognosis research, diverse machine learning models have applied to the problems of cancer susceptibility (risk assessment), cancer recurrence (redevelopment of cancer after resolution), and cancer survivability, regarding an accuracy (or an AUC--the area under the ROC curve) as a primary measurement for the performance evaluation of the models. However, in order to help medical specialists to establish a treatment plan by using the predicted output of a model, it is more pragmatic to elucidate which variables (markers) have most significantly influenced to the resulting outcome of cancer or which patients show similar patterns.

**Methods:**

In this study, a coupling approach of two sub-modules--a predictor and a descriptor--is proposed. The predictor module generates the predicted output for the cancer outcome. Semi-supervised learning co-training algorithm is employed as a predictor. On the other hand, the descriptor module post-processes the results of the predictor module, mainly focusing on which variables are more highly or less significantly ranked when describing the results of the prediction, and how patients are segmented into several groups according to the trait of common patterns among them. Decision trees are used as a descriptor.

**Results:**

The proposed approach, 'predictor-descriptor,' was tested on the breast cancer survivability problem based on the surveillance, epidemiology, and end results database for breast cancer (SEER). The results present the performance comparison among the established machine leaning algorithms, the ranks of the prognosis elements for breast cancer, and patient segmentation. In the performance comparison among the predictor candidates, Semi-supervised learning co-training algorithm showed best performance, producing an average AUC of 0.81. Later, the descriptor module found the top-tier prognosis markers which significantly affect to the classification results on survived/dead patients: 'lymph node involvement', 'stage', 'site-specific surgery', 'number of positive node examined', and 'tumor size', etc. Also, a typical example of patient-segmentation was provided: the patients classified as dead were grouped into two segments depending on difference in prognostic profiles, ones with serious results with respect to the pathologic exams and the others with the feebleness of age.

## Background

Breast cancer is the second most lethal cancer in women (Lung cancer is the leading cancer killer in women), and represents 14.1% of all new cancer cases in the U.S. in 2013 [1,2]. The good news is that women are living longer with breast cancer. The 5-year survival rate for women diagnosed with breast cancer is 89.2% [2]. Due to early detection, increased accuracy in cancer prognosis, and better treatment options, breast cancer mortality rates declined by about 34% since 1990 [3]. Particularly, advances in cancer prognosis help both physicians and patients in establishing an adjuvant treatment plan [4-6]. A prognosis is an estimate of the likely course and outcome of a disease. The prognosis of a patient diagnosed with cancer is often viewed as the chance that the disease will be treated successfully and that the patient will recover. Cancer prognosis includes susceptibility (cancer risk assessment), recurrence (redevelopment of cancer after resolution), and survivability (survivability of a patient, life expectancy, progression, tumor-drug sensitivity, etc.) [7]. In this paper, we scope the last one, survivability: whether a patient is to be or not to be a survivor after 1,825 days (5 years) from the date of cancer diagnosis.

Diverse predictive models from machine learning or data mining have employed to perform predictions on survivability. In [6], the authors conducted a wide ranging investigation of different machine learning methods, discussing issues related to the types of data incorporated and the performance of these techniques in breast cancer prognosis. This review provides detailed explanations leading to first-rate research guidelines for the application of machine learning methods during cancer prognosis. The authors of [5] used two popular data mining algorithms, artificial neural networks (ANN) and decision trees (DT), together with a common statistical method, logistic regression, to develop prediction models for breast cancer survivability. The DT was shown to be the best predictor. An improvement in the results of DT for the prognosis of breast cancer survivability is described in [4]. The authors propose a hybrid prognostic scheme based on weighted fuzzy decision trees. This hybrid scheme is an effective alternative to crisp classifiers that are applied independently. In [8], the authors conducted data preprocessing with RELIEF attribute selection and used the Modest AdaBoost algorithm to predict breast cancer survivability. The study used the Srinagarind hospital database. The results showed that Modest AdaBoost performed better than Real and Gentle AdaBoost. They then proposed a hybrid scheme to generate a high quality data set to develop improved breast cancer survival models [9]. In [10], support vector machines (SVM) based classification was carried out concerning both the prognosis and diagnosis problems of breast cancer. From the comparison results with ANN and Bayesian method, they demonstrates the superiority of SVM in terms of sensitivity, specificity and accuracy.

In prediction of survival of breast cancer patients, the performance of the established machine learning models including ANN, SVM, semi-supervised learning (SSL), Bayesian Methods, etc., has been often compared and the winner model is renewed paper by paper [4-6,8-11]. While such studies have been devoted to enhancement of the predictive power or accuracy of the predictive model, interpretability of the predicted results has received less attention. Most of them are like a black-box module only producing the prediction results and accuracy as a measure of performance for comparison. In other words, it is difficult to know what happened during prediction and how we obtained the results: for instance, the questions like 'which factors(variables) are most significantly contributed to survival/death classification?' or 'are there subgroups of patients that show a similar pattern?' are usually veiled. In practice, however, the answers for those questions benefits for medical practitioners and patients in many ways. By knowing the significant factors, we can make a proper choice of therapy, which may elevate the likelihood of successful treatments. At the same time, redundant or unimportant factors for breast cancer can be ruled out from then on, which will lead to reduction in time and cost during data collection and during treatment as well. On the other hand, patient segmentation (tying similar patients as a group) also used to help determine whether adjuvant treatments should be given to a particular patient, i.e., a doctor can decide whether a patient who has had surgery may benefit from a certain type of chemotherapy. The segmentation results based on the trait of common patterns of the patients can help predict how aggressive a patient's cancer may be and how well the cancer may respond to certain types of drugs. Among the representatives in machine learning models, a DT is a model equipped with reasonably good general ability and interpretability [5,12-14]. However, it would occur that its performance does not reach to those of the up-to-date models, i.e., SVM, SSL, Bayesian Methods [15,16]. To investigate the predicted results further, one may not want to simply give up using the winner model.

To circumvent the dilemma, we suggest a coupling approach of two sub-modules--a predictor and a descriptor. The predictor module generates the predicted output for cancer survivability, and any relevant predictive model can be employed as a predictor such as SVM, ANN, SSL and Bayesian method, etc. In the current study, we use the SSL based Co-training algorithm which showed outperformance than others in the previous study [11]. The model generates pseudo-labels by co-training multiple SSL member models, which assign them to unlabeled data before treating them as if they were labeled. As the labeled data increase, the predictive performance of the ordinary SSL increases. The algorithm realizes the tenet of 'the more labeled data, the better prediction' which would be applied to most machine learning algorithms. On the other hand, the descriptor module post-processes the results of the predictor module, and provides variable importance and patient segmentation: which variables are more highly or less significantly ranked when describing the results of the prediction, and how patients are segmented into several groups according to the trait of common patterns, respectively. Decision trees (DT) are used as a descriptor.

The rest of this paper is organized as follows. Section 2 presents the proposed 'predictor-descriptor' approach. In the predictor module, SSL Co-training is explained following a brief introduction to SSL. In the descriptor module, variable importance and patient segmentation are described. Section 3 shows the experimental results of the proposed method on the surveillance, epidemiology, and end results (SEER) cancer incidence database, which is the most comprehensive source of information on cancer incidence and survival in the USA [2]. Performance comparison of SSL Co-training and the latest machine learning models, and interpretations with clinical implications on the results are provided. In Section 4, we present our conclusions.

## Proposed method: a coupling approach of a predictor and a descriptor

**Figure 1:**
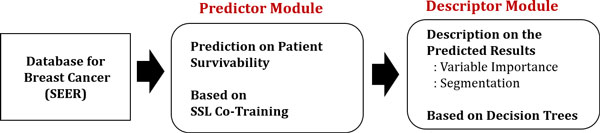
**Schematic description on the procedure of the proposed method**. Schematic description on the procedure of the proposed method.

The proposed model is designed through two phases of modeling: a predictor and a descriptor. The predictor module generates the predicted labels for patient samples on whether the patient will be survived or not. SSL Co-training is employed as a model for the predictor module [11]. After then, the descriptor module post-processes the prediction results by using decision trees [12-14]. It profiles the reasons which variables are most determinant in identifying survived/dead patients, which translates as variable importance. The descriptor module also provides segmented results. A set of patients with similar values in variables is called a segment. By segregating the survived patients into homogeneous segments, a medical expert now can tailor a proper investigative and treatment plan for each segment. Figure 1 presents the overall procedure of the proposed method.

### The predictor module: semi-supervised learning and SSL co-training

In many real applications, there is a large supply of unlabeled data, and we can collect them with only a little effort in general. However, we may not be able to obtain enough labeled data since it is often costly, difficult, or time consuming to generate the labels for data [17,18]. Recently, many machine learning researchers have found that unlabeled data, when used in conjunction with a small amount of labeled data, can produce considerable improvement in learning accuracy. And it is "paradigmed" as semi-supervised learning or simply SSL. SSL exploits the knowledge of the input structure from unlabeled data and at the same time utilizes the label information provided by labeled data.

SSL may be a good candidate to use a predictive model for cancer survivability, particularly when the available dataset for model learning has an abundance of unlabeled patient cases but a lack of labeled ones. Like many other machine learning algorithms, however, the availability of more labeled data leads to better performance. This motivated our previous work, SSL Co-training [11]. The proposed model generates "pseudo-labels" and it increases the performance of SSL. The model is based on graph-based SSL [19,20].

**Figure 2:**
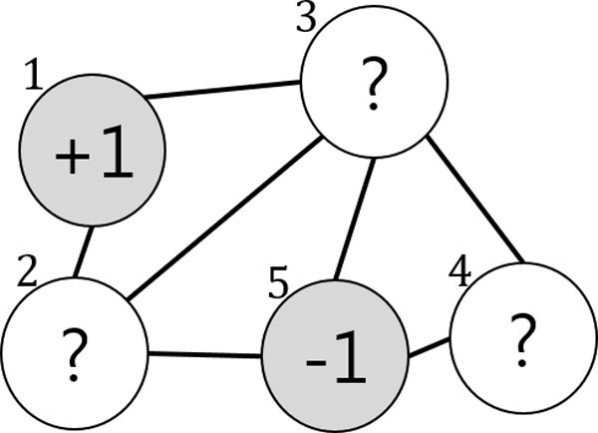
**Original SSL Graph**. In graph-based SSL, the labeled nodes are represented by '+1(survived)' and '-1(dead)', whereas unlabeled nodes are represented by '?'.

In graph-based SSL, a weighted graph is constructed where the nodes represent the labeled and unlabeled data points while the edges reflect the similarity between data points. Figure 2(a) depicts a graph with two labeled and three unlabeled data points. Given $n\left(=n_{l}+n_{u}\right)$ data points from the sets of labeled $L=\left\{{\left(x_{i},y_{i}\right)}_{i=1}^{n_{l}}\right\}$ and unlabeled $U=\left\{{\left(x_{j}\right)}_{j=n_{l}+1}^{n}\right\}$, the labeled nodes are set to $y_{l}\in \left\{-1,+1\right\}$, while the unlabeled nodes are set to zero $\left(y_{u}=0\right)$. The edges between the two nodes $x_{i}$ and $x_{j}$ are usually measured by the Gaussian function

(1) $$w_{ij}=\left\{\begin{array}{cc} \text{exp}\left(-\frac{{\left(x_{i}-x_{j}\right)}^{T}\left(x_{i}-x_{j}\right)}{\alpha^{2}}\right) & if\;i\sim j\\ 0 & otherwise \end{array}\right\}$$

where i ~ j indicates that the two nodes are connected, and the value of the similarity is represented by a matrix $W=\left\{w_{ij}\right\}$. Then the label information can propagate from (labeled) node $x_{i}$ to node (unlabeled) node $x_{j}$ when they are coupled by a path of high density (e.g., the value of $w_{ij}$ is large), their outputs are likely to be close, whereas their outputs need not be close if they are separated by a low-density region (e.g., the value of $w_{ij}$ is small) [15,16,19,20].

The algorithm will output an n-dimensional real-valued vector $f={\left[f_{l}^{T}\;f_{u}^{T}\right]}^{T}={\left(f_{1},\ldots ,f_{l},f_{l+1},\ldots ,f_{n=l+u}\right)}^{T}$, which can generate a threshold value to perform the label predictions on $\left(f_{1},\ldots ,f_{n}\right)$ as a result of the learning. There are two assumptions: a loss function ($f_{i}$ should be close to the given label of $y_{i}$ in labeled nodes) and label smoothness (overall, $f_{i}$ should not be too different from $f_{i}$ for the neighboring nodes). These assumptions are reflected in the value of f by minimizing the following quadratic function [17,21,22]:

(2) $$\underset{f}{\text{min}}{\left(f-y\right)}^{T}\left(f-y\right)+\mu f^{T}Lf,$$

where $y={\left[y_{1},\ldots ,y_{l},0,\ldots ,0\right]}^{T}$ and the matrix  L, which is known as the graph Laplacian matrix, is defined as L=D-W where $D=diag\left(d_{i}\right)$, $d_{i}=\underset{i}{\sum }w_{ij}$. The parameter  μ trades off loss and smoothness. Thus, the solution of this problem becomes

(3) $$f={\left(I+\mu L\right)}^{-1}y.$$

Based on the basic framework of graph-based SSL, SSL Co-training obtains more labeled data by assigning labels to unlabeled data, i.e., "pseudo-labels," and uses them for model learning as if they were labeled. The model involves multiple member models where pseudo-labels are determined based on agreements among the members. Therefore, it is named as SSL Co-training [11]. The toy example shown in Figure 1 is helpful for understanding the model.

**Figure 3:**
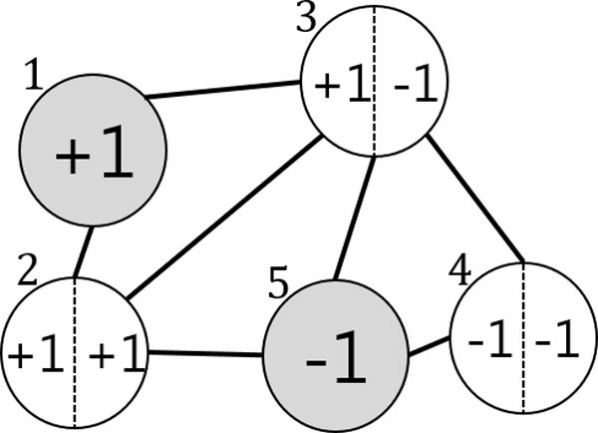
**SSL Co-training: Predicted labels**. The two figures 2 and 3 provide schematic description of SSL Co-training. At the start of the algorithm, each of the member models (for simplicity, we assume two classifiers) is trained on the original graph in Figure 2.

**Figure 4:**
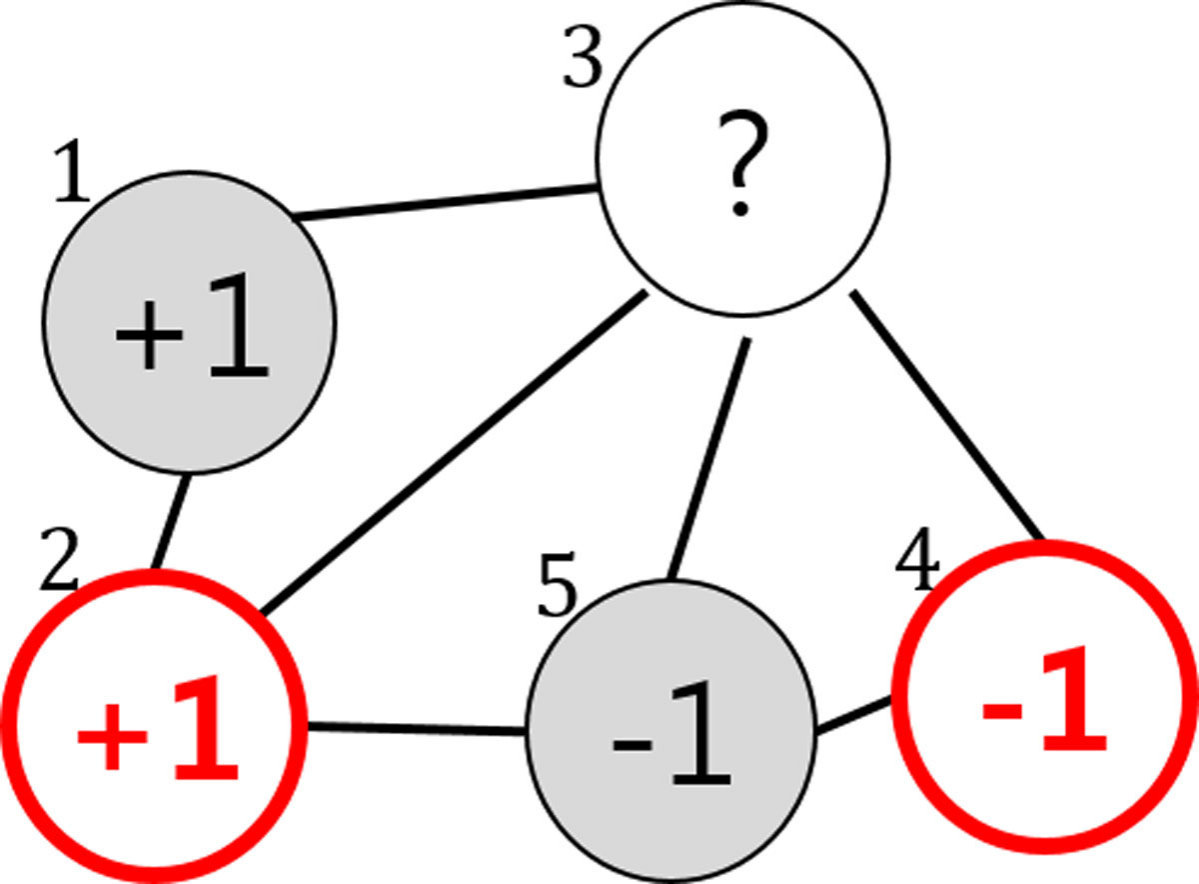
**SSL Co-training: Pseudo labeled graph**. After training, both member models produce predicted labels for the unlabeled nodes. The unlabeled nodes are pseudo-labeled when the member models agree on labeling, or it remains unlabeled. The resulting graph is shown in Figure 4.

In the beginning Figure 2, the two data points $x_{1}$ and $x_{5}$ belong to the labeled set $L=\left\{\left(x_{1},+1\right),\left(x_{2},-1\right)\right\}$ and the labels are given as $y_{1}=+1$ and $y_{5}=-1$, respectively. $x_{2}$, $x_{3}$, and $x_{4}$ belong to the unlabeled dataset $U=\left\{x_{2},x_{2},x_{3}\right\}$. For simplicity, we assume that two member models, $F_{1}$ and $F_{2}$, are provided (more concretely, two SSL classifiers) and that they are independent. At the start of the algorithm, each of the two classifiers is trained on  L and  U following the objective function in (2) as an ordinary SSL. After training (iteration 1) in Figure 3, the predicted labels for the three data points are given by $F_{1}$ and $F_{2}$. For $x_{2}$, the two classifiers agree on labeling $y_{2}^{1}=y_{2}^{2}=+1,$ so its pseudo-label becomes $y_{2}=1$. Likewise, $x_{4}$ obtains the pseudo-label $y_{4}=-1$. However, the two members disagree on the labeling of $x_{3}:y_{3}^{1}=+1$ but $y_{3}^{2}=-1$. Therefore, it remains unlabeled. In the next iteration (iteration 2) in Figure 4, the labeled dataset is increased by the two pseudo-labeled data points $L=\left\{\left(x_{1},+1\right),\left(x_{2},+1\right),\left(x_{3},-1\right),\left(x_{4},-1\right)\right\}$, and the unlabeled data set is decreased to $U=\left\{x_{3}\right\}.$ Similar to the previous iteration, $F_{1}$ and $F_{2}$ provide $x_{3}$ with the predicted labels $y_{3}^{1}=+1$ and $y_{3}^{2}=-1$, respectively. However, they still fail to agree on the labeling of $x_{3}$. This leads to the same result as the previous iteration, so the iteration stops. SSL Co-training increases the performance of an ordinary SSL thanks to the pseudo-labeled data points. Further details on the method can be found in [11].

### The descriptor module

In the descriptor module, we obtain information on the importance of the variables used as input for the survivability prediction, and the detailed segmentation for the patient groups. The patient samples with predicted labels by SSL Co-training are fed to decision trees (DT) and re-classified. A DT is a traditional supervised learning model, and its general ability is known to be reasonably good [5,12-14]. This implies a DT itself can be used as a predictive model for the breast cancer survivability. However, since there are more recent and sophisticated models with a better performance such as SVM and SSL (the comparison results are provided in the following experiment section), we opt to use a DT as a post-processor for describing the results of those predictive models. DT can provide interpretability on what happened in the predictor module: for instance, 'which variables are most significantly contributed to survival/death classification?' and 'are there subgroups of patients that show a similar pattern?', etc. The answers for the questions are naturally obtained by reclassifying the (variables, the predicted label) pairs of patient samples. Note that neither a validation nor a test set is used since the DT here is only employed for the purpose of description on the output of the predictor module, not for prediction. During the training, DT recursively splits samples in a root node into two or more subgroups until a final tree is constructed. While a tree is growing, it identifies a splitting variable and corresponding threshold value that maximizes the homogeneity of the resulting two or more subgroups of samples.

The issue of variable importance is related to the splitting criteria of DT. The most well-known criteria includes Gini index (used in CART) [13], Entropy based information gain (used in ID3, C4.5, C5) [12], and Chi-squared test (used in CHAID) [14]. There are some differences among those criteria, the commonly used measure of importance is based on the surrogate splits ${\tilde{s}}_{x}$ computes the improvement in homogeneity by the splitting of variable  x, $\Delta I\left({\tilde{s}}_{x},t\right)$, at each ynode  t in the final tree, t∈T. Then, the measure of importance M(x) of variable × is defined as the sum across all splits in the tree of the improvements that  x has when it is used as a primary or surrogate splitter [13,23]:

(4) $$\text{M}\left(x\right)=\underset{t\in T}{\sum }\Delta I\left({\tilde{s}}_{x},t\right).$$

Since only the relative magnitudes of the M(x) are interesting, the actual values of variable importance are the normalized quantities. The most important variable then has value 1, and the others are in the range 0 to 1.

(5) $$\text{VI}\left(x\right)=\frac{\text{M}\left(x\right)}{\underset{x}{\text{max}}\;\text{M}\left(x\right)}$$

**Figure 5:**
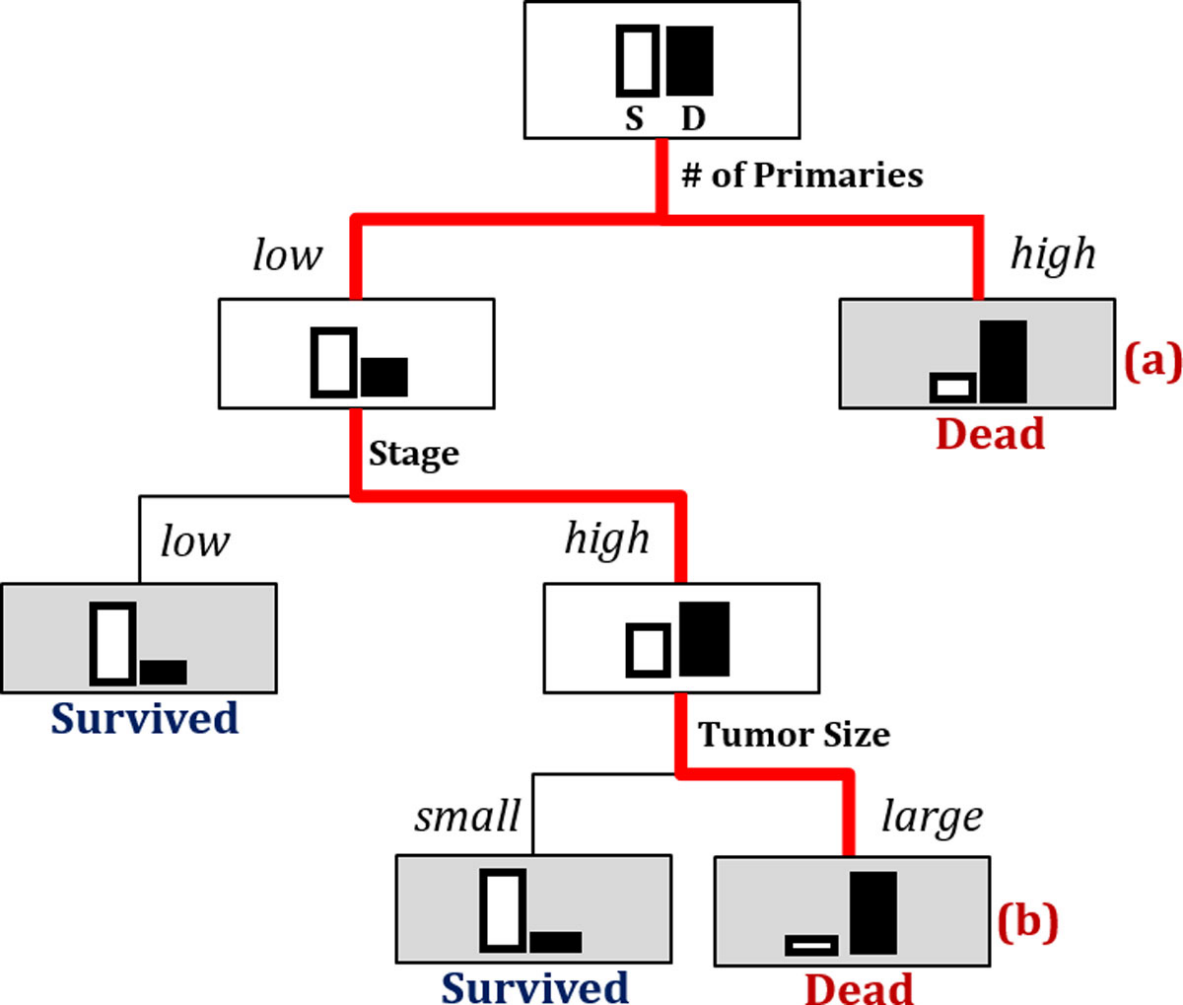
**Using a decision tree to obtain variable importance and segmentation by reclassifying the results of the predictor module**. Using a decision tree to obtain variable importance and segmentation by reclassifying the results of the predictor module.

Figure 5 exemplifies a final tree after re-classification. The leaf (shaded) nodes are labeled as either survived or dead. One can figure out which variables contributed significantly for the splitting by tracing down the tree from the root node to the leaf. Generally, a variable in a higher level is regarded as more important than the one in a lower level. But it should be noted that those variables that, while not giving the best split of a node, may give the second or third best are often hidden in the final tree. For instance, if classification accuracies of two variables $x_{1}$ and $x_{2}$ are similar, assuming $x_{1}$ is slightly better than $x_{2}$, then the variable $x_{2}$ may never occur in any split in the final tree. In such a situation, we would require the measures in Eq.(4) and Eq.(5), the variable importance based on surrogate split, to detect the importance of $x_{2}$.

On the other hand, the issue on patient segmentation is related to finding a route from the root node to a leaf node in the resulting tree. From the binary classification results of the predictor module, we only know difference between the two groups of the survived patients and of the dead. In practice, however, we may want to know further. Looking into the records of the patients who are predicted to be dead (or survived), for instance, there may be several different reasons or patterns which lead them to death (or survival). The segmentation on patients depending on difference in patterns can be obtained from the resulting tree. Figure 5 shows a toy case: the patients who are predicted to be highly likely to be dead are now segregated into two segments: (a) the ones with a very high in 'Number of Primaries' and (b) the others with a low in 'Number of Primaries' but a high in 'Stage' and a large in 'Tumor Size'. Depending on the trait of the segment, one can tailor an appropriate medical plan and action.

## Experiments

### Data

In this study, Surveillance, Epidemiology, and End Results data (SEER, 1973-2003) is used for the experiment. SEER is an initiative of the National Cancer Institute and the premier source for cancer statistics in the United States and claims to have one of the most comprehensive collections of cancer statistics [2,7]. The data consists of 162,500 records with 16 input variables and one target class variable. It includes incidence, mortality, prevalence, survival, lifetime risk, and statistics by race/ethnicity. Particularly, survivability of patients with breast cancer depends on two different types of prognostic variables: 1) chronological (indicators of how long the cancer has been present, e.g. tumor size) and 2) biological (indicators of metastatic aggressive behavior of a tumor, e.g. tumor grade) [24]. They determine, either or not a particular tumor might respond to a specific therapy. The 16 input variables are tumor size, number of nodes, number of primaries, age at diagnosis, number of positive nodes, marital status, race, behavior code, grade, extension of tumor, node involvement, histological Type ICD, primary site, site-specific surgery, radiation, and stage. The target variable 'survivability' is a binary categorical feature with values '-1' (not survived or dead) or +1 (survived). Table 1 summarizes the variables and the corresponding descriptions.

**Table 1:** Prognostic elements of breast cancer survivability (SEER).

Prognostic elements			Description	Number of distinct values / mean ± std.dev
**Discrete** **Variables**	1	**Race**	Ethnicity: White, Black, Chinese, etc.	16
	2	**Radiation**	None, Beam Radiation, Radioisotopes, Refused, Recommended, etc.	6
	3	**Primary Site**	Presence of tumor at particular location in body. Topographical classification of cancer.	9
	4	**Histological Type**	Form and structure of tumor	30
	5	**Behavior Code**	Normal or aggressive tumor behavior is defined using codes.	2
	6	**Grade**	Appearance of tumor and its similarity to more or less aggressive tumors	5
	7	**Site Specific Surgery**	Information on surgery during first course of therapy, whether cancer-directed or not.	12
	8	**Stage**	Defined by size of cancer tumor and its spread	10
	9	**Clinical Extension of tumor**	Defines the spread of the tumor relative to the breast	16
	10	**Lymph Node** **Involvement**	None, (1-3) Minimal, (4-9) Significant, etc.	7
	11	**Marital Status**	Married, Single, Divorced, Widowed, Separated	4

**Continuous Variables**	12	**Age at Diagnosis**	Actual age of patient in years	63.64 ± 14.25
	13	**Tumor Size**	2-5 cm; at 5 cm, the prognosis worsens	116.78 ± 286.64
	14	**Number of Positive** **Nodes Examined**	When lymph nodes are involved in cancer, they are known as positive.	27.29 ± 42.26
	15	**Number of Nodes** **Examined**	The total number of (positive/negative) lymph nodes that were removed and examined by the pathologist.	13.61 ± 17.49
	16	**Number of Primaries**	Number of primary tumors (1-6)	0.54 ± 1.29

**Survivability**			Target binary variable defines class of survival of patient.	

### Results of the predictor module

In the predictor module, the generalization abilities of five representative predictive models, i.e., DT, ANN, SVM, SSL, and SSL-Co training, were compared. The area under the receiver operating characteristic (ROC) curve (AUC) was used as performance measures [25]: the AUC assess the overall performance of a classification model, which is a threshold-independent measure based on the ROC curve that plots the tradeoffs between sensitivity and 1−specificity for all possible values of threshold. The breast cancer survival dataset contains 128,469 positive cases and 34,031 negative cases. To avoid the difficulties in learning of the predictive models, caused by the large-sized and class-imbalanced dataset, 40,000 data points were used for the training set and 10,000 for the test set, which were drawn randomly without replacement. The equipoise dataset of 50,000 data points was eventually divided into ten groups and five-fold cross validation was applied to each.

The model parameters were searched over the following ranges for the respective models. For DT, CART was employed with default setting [13]. It is a non-parametric model, and hence it did not necessarily require parameter searching. For ANN, the number of 'hidden nodes' and the 'random seed' for the initial weights were searched over hidden-node = {3, 6, 9, 12, 15} and random-seed = {1, 3, 5, 7, 10} [26]. For SVM, the values for the RBF kernel width 'Gamma' and the loss penalty term 'C' were selected by searching the ranges of C = {0.2, 0.4, 0.6, 0.8, 1} and Gamma = {0.0001, 0.001, 0.01, 0.1, 1} [27]. For the SSL and SSL Co-training models, the values for the number of neighbors 'k' and the tradeoff parameter 'Mu' between the smoothness condition and loss condition in (1) were searched over k = {3, 7, 15, 20, 30} and Mu = {0.0001, 0.01, 1, 100, 1000}, respectively.

**Figure 6:**
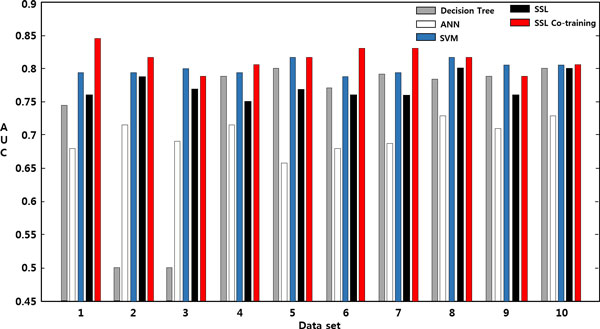
**Performance (AUC) comparison over 10 data sets**. Performance (AUC) comparison over 10 data sets: DT, ANN, SVM, SSL, and SSL Co-training.

Table 2 shows a comparison of the results with DT, ANN, SVM, SSL, and SSL Co-training in terms of the AUC. For each of the five models, the best performance was selected by searching over the respective model-parameter space. SSL Co-training produced an average AUC of 0.81, which was the best of the five models although comparable performance was delivered by SVM. On the other hand, DT showed an average AUC of 0.73, and just ranked the worst performed model ANN. Either DT or ANN may be a good predictive model for some other problems, but are less likely to be the one than other three models in the current study. Figure 6 visualizes the AUC performance of the five models using bar graphs.

**Table 2:** Performance (AUC) comparison of the five predictive models

Data Set	1	2	3	4	5	6	7	8	9	10	**Avg**.
**DT**	0.74	0.50	0.50	0.78	0.80	0.82	0.79	0.77	0.78	0.80	**0.73**
**ANN**	0.68	0.72	0.68	0.72	0.66	0.68	0.67	0.73	0.71	0.73	**0.70**
**SVM**	0.79	0.79	0.80	0.79	0.82	0.78	0.79	0.82	0.81	0.81	**0.80**
**SSL**	0.77	0.79	0.78	0.76	0.78	0.77	0.77	0.8	0.78	0.8	**0.78**
**SSL Co-training**	0.84	0.82	0.80	0.81	0.82	0.84	0.83	0.82	0.78	0.81	**0.81**

### Results of the descriptor module

For each of the 10 data sets, the test samples with predicted labels were input to the descriptor module together with the training samples. The labels for the test samples were obtained from SSL Co-training, which performed best among the five competing predictive models.

**Figure 7:**
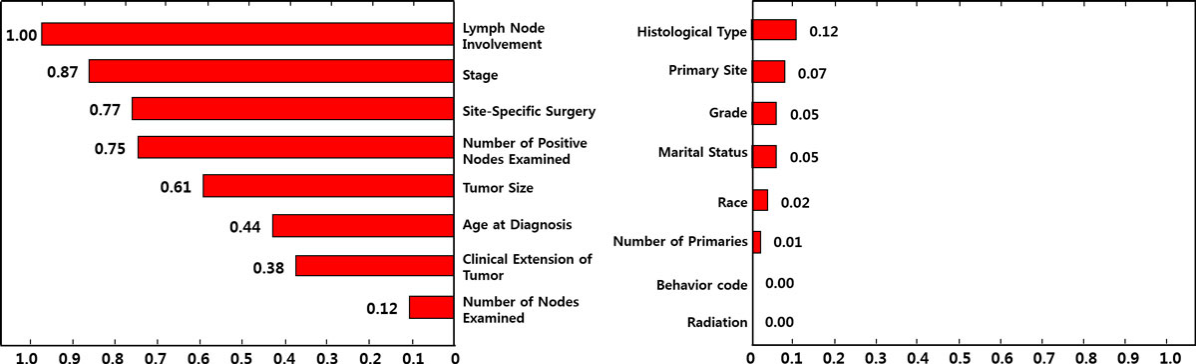
**Variable Importance**. Variable importance: the 16 input variables are ranked by the order of variable importance Eq.(5).

Figure 7 shows ranking of the 16 input variables in terms of Eq.(5), the relative magnitudes to the value of the most important variable. It shows that 'Lymph Node Involvement' is the most determinant variable in identifying survived/dead patients, therefore it has a value of 1. And in order of variable importance, 'Stage', 'Site-specific Surgery', 'Number of Positive Nodes Examined' and 'Tumor Size' belong to the top-tier ranked up to 5th variables, and are regarded as more important ones than the rest.

The five variables are all related to the findings from a pathologic exam. It is known as the best way to assess lymph node status and can give a first estimate of breast cancer stage and the size of tumor. Usually, a surgeon removes some lymph nodes in the armpit with a technique called sentinel node biopsy. Then, a pathologist studies these nodes under a microscope. As part of the initial work-up or first course of therapy, a surgical procedure that removes and/or destroys tissue of the specific-site performed. Prognosis is poorer when cancer has spread to the lymph nodes (lymph node-positive). The more lymph nodes that contain cancer, the poorer the prognosis tends to be. Non-invasive and early stage invasive breast cancers have a better prognosis than later stage cancers. And, the poorest prognosis is for metastatic breast cancer, where the cancer has spread beyond the lymph nodes to other parts of the body. Enlarged nodes can be a sign of cancer spread [28].

**Figure 8:**
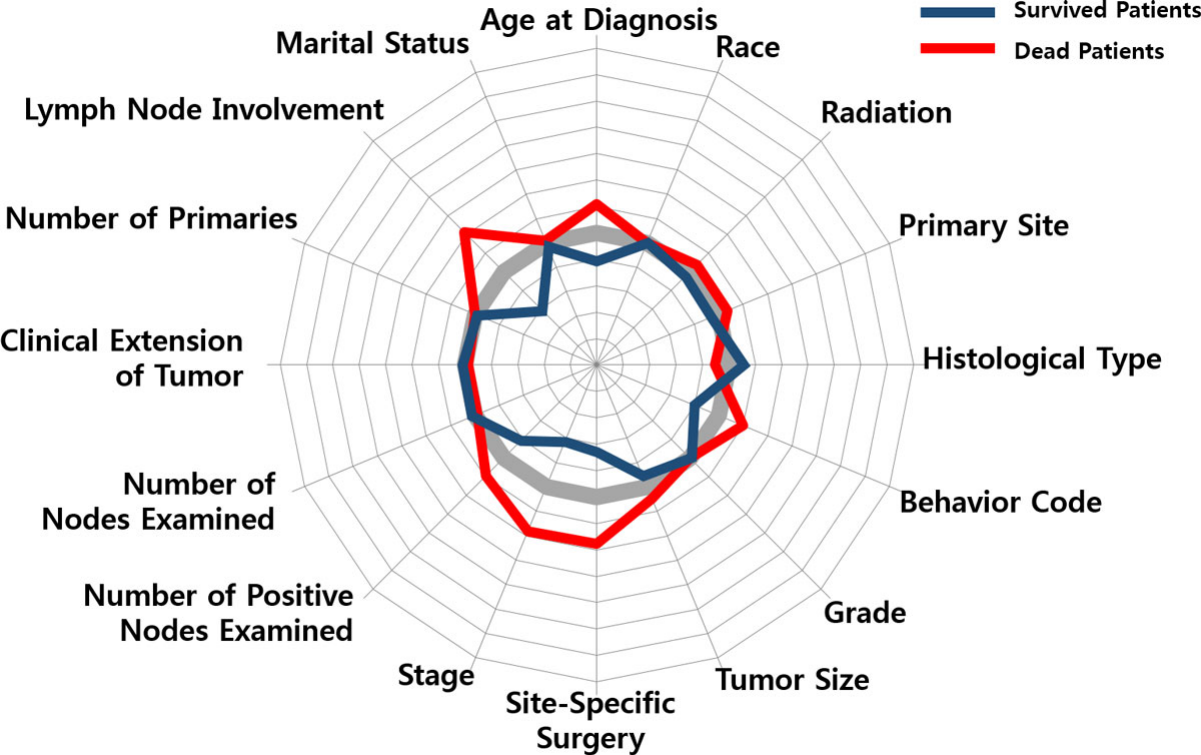
**Variable Profiling**. Variable profiling: average values of the 16 variables for (a) survived patients and (b) dead patients.

To discern that how those variables influence to classification, the two patient groups of the survived and the dead were profiled, respectively. Figure 8 shows a radial diagram of the average values of 16 variables for the two patient groups. Each axis of the diagram represents a scaled value relative to its range. The grey line in the center stands for the overall average of the variable, whereas the blue/red line stands for the group average of the survived/dead.

Compared the averages of the two groups, significant differences can be found by 'Lymph Node Involvement', 'Number of Positive Nodes Examined', 'Stage', 'Behavior Code', 'Site-Specific Surgery', 'Tumor Size', 'Age at Diagnosis', whereas 'Marital Status' and 'Race' do not provide significant information on discriminating the two groups. Relatively, a general pattern of the survived patients is less involvement of lymph nodes, an earlier stage, a smaller sized tumor, non-invasive in cancer behavior, less (site-specific) surgeries, younger in terms of age at diagnosis. On the other hand, the dead patients show a pattern of larger spread of cancer over lymph nodes, a larger tumor size, more aggressive and invasive cancer behavior, more surgeries and radiation therapies, and an older age at diagnosis.

**Figure 9:**
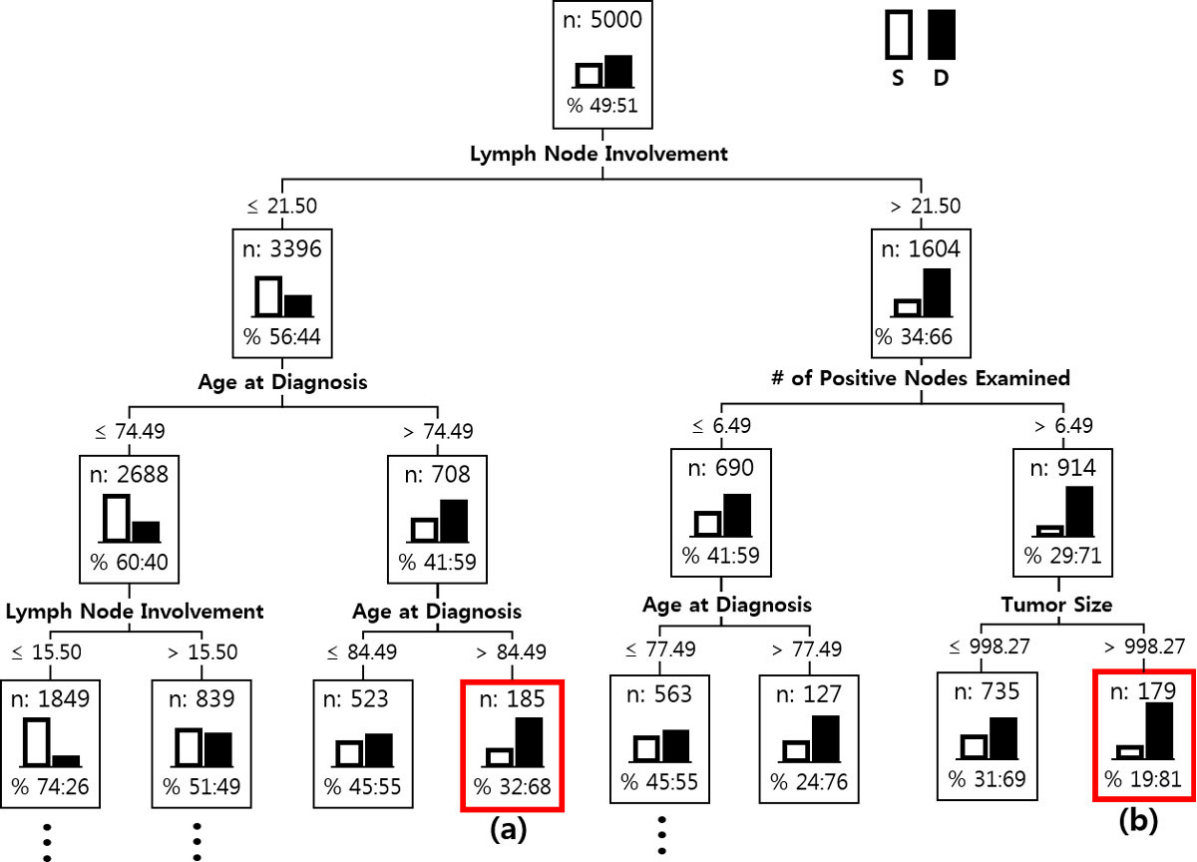
**Patient Segmentation**. Patient Segmentation: The first three levels of the resulting decision tree. In a node, the proportion of the survived and the dead are represented as the white bar and the black bar, respectively.

The results of the predictor module were further examined by segmenting the survived/dead patients into several subgroups using DT. Figure 9 shows the first three levels of the resulting tree. (The complete tree has six levels with 15 leaf nodes.) The tree splits the root node of the 5,000 patients into several children nodes by successively choosing the most significant variables in classifying the patients into the survived/dead. A variable in a higher level of the tree is more important than the one in a lower level. Similar results were obtained as in variable importance: 'Lymph Node Involvement', 'Number of Positive Nodes Examined', 'Age at Diagnosis', 'Stage', and 'Tumor Size' were used as early splitters of the tree, and in a full tree, 'Grade', 'Site-Specific Surgery', 'Number of Node Examined', and 'Primary Site', etc. were additionally included. As the tree grows, the purity at the leaf nodes measured by the proportion of patient assigned to the dominant class (either the survived or the dead) increases. In a node, the proportion of the survived/dead are represented as a histogram, the white bar is for the survived and the black one is for the dead. A leaf node in the resulting tree is called a segment of the patients who are similar in their prognosis factors. The segment profiling for a leaf node is determined by the variables (with the corresponding values) that contributed significantly for the node-split by tracing back the tree from the leaf node to the root. In the tree, there are many leaf nodes and each of them has different profiling, and therefore the patients who are classified into a same class (either survived or dead) in the predictor module are further segregated into several segments in the description module depending on which leaf nodes they belong to.

In Figure 9, two typical cases of patient segments, (a) and (b), are marked with the red-outlined boxes. Both belong to the class of the dead, but show different reasons. The following two radial diagrams in Figure 8 illustrate the difference.

**Figure 10:**
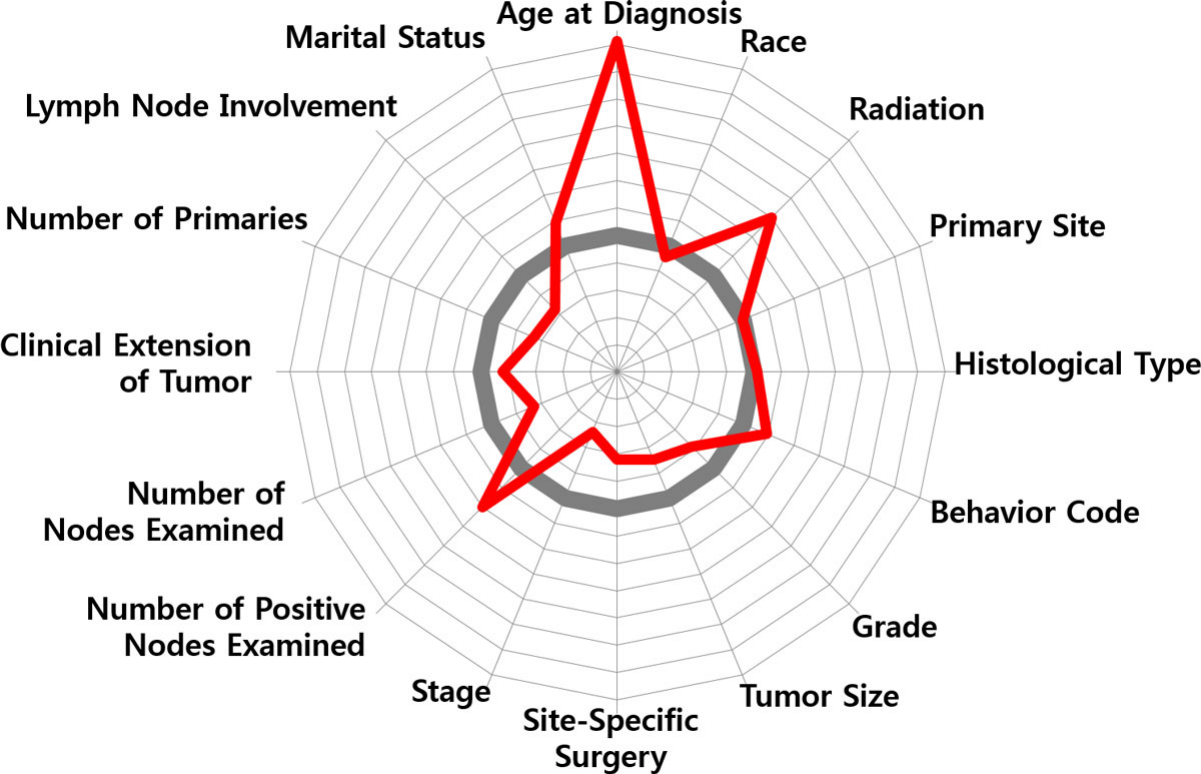
**Patient Segmentation: Feebleness of age**. Patient Segmentation: Two radial diagrams in Figure 10 and 11 illustrate difference of patient segments in terms of patterns of prognosis factors.

**Figure 11:**
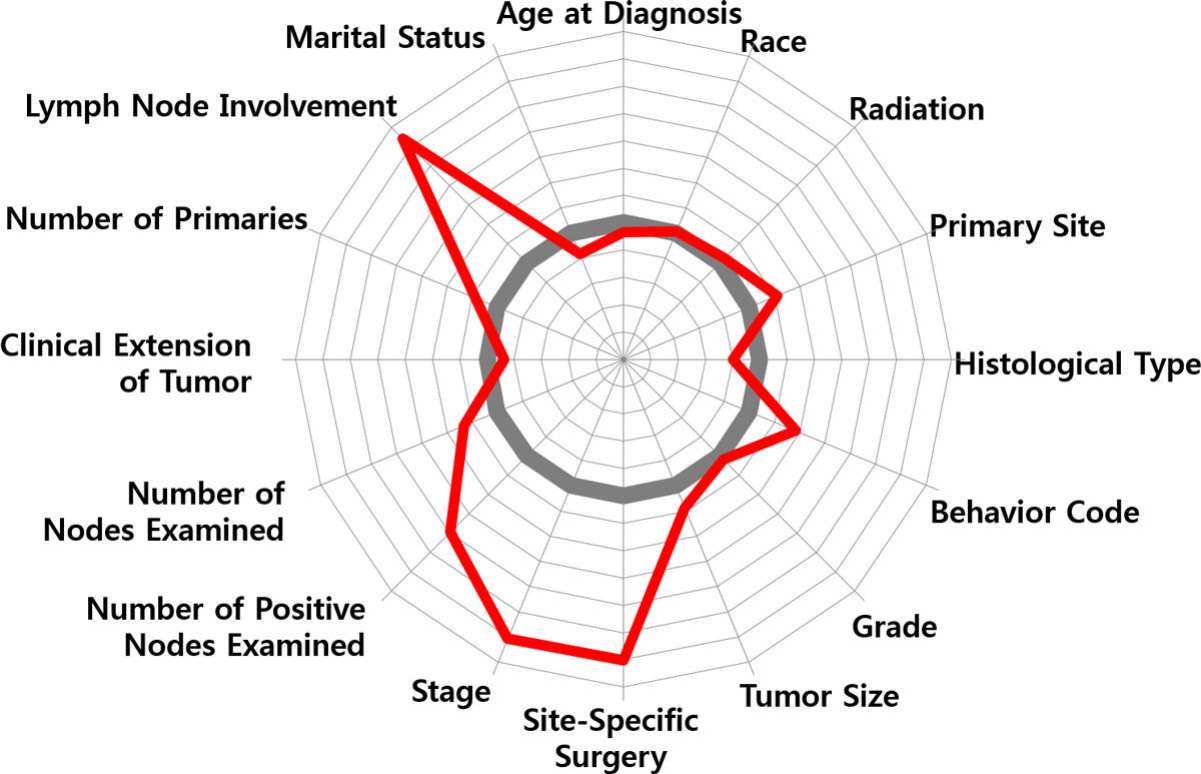
**Patient Segmentation: A serious pattern with respect to the pathologic results**. Patient Segmentation: Two radial diagrams in Figure 10 and 11 illustrate difference of patient segments in terms of patterns of prognosis factors.

Compared with the averages of the dead patients in Figure 8, the patient segment in Figure 10 shows a different pattern: low in 'Lymph Node Involvement', small in 'Tumor Size', early in 'Stage', low in 'Site-Specific Surgery', high in 'Radiation', but a very high peak in 'Age at Diagnosis'. One may make a mere conjecture that those patients had not been so serious from the viewpoint of the pathologic exam. Then, the main factor that had driven them to death might have been the feebleness of age (they are over age of 84). On the contrary, the patient segment in Figure 11 shows a serious pattern with respect to the pathologic results: a high peak in 'Lymph Node Involvement', large in 'Number of Positive Nodes Examined', late in 'Stage', aggressive and invasive in 'Behavior Code' of cancer, a larger number in 'Site-Specific Surgery'.

Figure 10 and 11 only present a tip of the possibilities of patient segmentation. With a more abundance of cancer prognosis factors, the presence of detailed segmentation can help predict the chances for long-term survival of the patients and also guide proper treatments that fit for each of the segments.

## Conclusion

In this study, we proposed a model that predicts the survivability of the breast cancer patients and provides the interpretations on the results in terms of cancer prognosis factors. The model is composed of two modules, a predictor and a descriptor. The predictor module of the model classifies patients into two classes of the survived and the dead, and then the descriptor module calculates the importance of prognosis factors, and groups the predicted patients with similar prognostic profiles. There are three noteworthy features of the proposed method. First, since the aim of the predictor module is to obtain the best prediction, it was designed to be flexible so that any winner model can be employed among the up-to-date machine learning algorithms. In this study, we used SSL Co-training which had been well validated in the authors' previous research [11]. Second, although the predictor module offers the best prediction, it seldom provide explicit explicability of which variable is the most significant during prediction. To unveil the implicit mechanism of the prediction procedure, variable importance calculation was embedded in the descriptor module. Knowing the significant variables will lead to better insights in cancer prognosis, and less time and cost by excluding redundant ones during data collection. In addition, the segmentation based on the decision trees was also integrated into the descriptor module. This can assist medical experts for the further investigation on prognosis factors and for the tailored treatment design according to unique features of the patient segments.

The present study triggers possible future works. First, the coupling approach of a predictor and a descriptor is yet general and its full application for different cancer types will still require a continued refinement, as well as broadening the number of prognosis factors whose cancer-specific ones are then selectively included. Second, from a pragmatic perspective, the importance ranking for cancer prognosis factors and the patient segments require practical checking by medical specialists. To incorporate this procedure systematically, an interactive mode which reflects intervention from users should be studied further.

Through this work, we would like to remark the followings reflecting reviewer's comment. In most studies which have applied a prediction algorithm to the medical domain problems, it is more or less missing that why the algorithm obtains the performance or how clinicians can put the obtained results into practice. Although the novelty of applied algorithms may only matter big to informaticians, but to clinicians, which algorithm is more novel or performs better than the other may not be the only concern. Rather, they need more kindness so that they better understand what happened in the prediction algorithm and its usability to the domain afterwards. This blind spot in informaticians' approaches to medical domain drives the motivation of this work. To take a broad view of this work, its value lies in that it is not only concerns the performance in prediction but also aware of the importance of description that raises clinicians' comprehension and practical usability of the method to the domain.

## Competing interests

The author declares that they have no competing interests.

## Authors' contributions

HS designed the idea, wrote the manuscript, and supervised the study process. YHN analysed the data and implemented the system. All authors read and approved the final manuscript.

## Declarations

Publication for this article has been funded by National Research Foundation of Korea(NRF) grant funded by the Korea government(MSIP) (2010-0028631/2013R1A1A3010440)

This article has been published as part of *BMC Medical Genomics *Volume 7 Supplement 1, 2014: Selected articles from the 3rd Translational Bioinformatics Conference (TBC/ISCB-Asia 2013). The full contents of the supplement are available online at http://www.biomedcentral.com/bmcmedgenomics/supplements/7/S1.
